# The civic turn of immigrant integration policies in the Scandinavian welfare states

**DOI:** 10.1186/s40878-017-0052-4

**Published:** 2017-03-20

**Authors:** Karin Borevi, Kristian Kriegbaum Jensen, Per Mouritsen

**Affiliations:** 1 Södertörn University, Huddinge, Sweden; 2 Aarhus University, Aarhus, Denmark

**Keywords:** Immigrant integration, Civic integration, Scandinavia, Convergence, Public philosophy

## Abstract

This special issue addresses the question of how to understand the civic turn within immigrant integration in the West towards programs and instruments, public discourses and political intentions, which aim to condition, incentivize, and shape through socialization immigrants into ‘citizens’. Empirically, it focuses on the less studied Scandinavian cases of Sweden, Norway, and Denmark. In this introduction, we situate the contributions to this special issue within the overall debate on civic integration and convergence. We introduce the three cases, critically discuss the (liberal) convergence thesis and its descriptive and explanatory claims, and explain why studying the Scandinavian welfare states can further our understanding of the nature of the civic turn and its driving forces. Before concluding, we discuss whether civic integration policies actually work.

## Introduction

This special issue, which grew out of lively discussions in an ECPR Joint Sessions workshop in Warsaw in April 2015, addresses the broad question of how to understand the turn, within immigrant integration policies, towards programs and instruments, public discourses and political intentions, which aim to condition, incentivize, and shape through socialization, immigrants into ‘citizens’. Focusing on the less studied cases of Sweden, Norway and Denmark, it critically engages a growing body of research within migration studies, which has diagnosed a convergence – in policies, discourses, or both – of Western states towards a liberal or civic integrationist middle ground, beyond ‘national models’ of either ethno-nationalist assimilation or multiculturalism, and indeed beyond nationalism as a political force.

The liberal convergence thesis, associated with the work of Joppke (2007) and others (e.g., Goodman, 2010; Green, 2007; Müller, 2007), has been challenged in comparative and single case studies (e.g., Bonjour & Lettinga, 2012; Jensen, 2016; Meer & Modood, 2009; Mouritsen, 2013; Borevi, 2014) as well as theoretically. Appraisals of the ‘turn’ differ, with some authors diagnosing an “illiberal”, “Schmittean” liberalism (Orgad, 2015; Triadafilopoulos, 2011) of “identity” and Foucauldian discipline (Tebble, 2006; Joppke, 2010), while others see a more benign, would-be Habermasian constitutional patriotism (Müller, 2007; Goodman, 2014).^1^ The thesis involves a claim about driving forces and draws attention to external and systemic factors, such as globalizing markets and supranational judicial norms and institutions, while it downplays or refutes national models and path dependence explanations.

Yet, significant variation remains between European countries in the scope, types, location, and degrees of demandingness of civic integration requirements introduced, as well as in the normative arguments that politicians use to justify them (Goodman, 2012, 2014). Indeed, according to Goodman (2012, p. 665–667) Sweden (along with Finland, Portugal and Spain) is not part of the turn since the country has not introduced “civic integration, formalized tests, assessment of country knowledge, or mandatory courses”. However, as articles in this volume reveal, if we apply a different, more ideational understanding of civic integration, it is clear that Swedish legislators are not immune to discourses associated with this trend. Are such remaining differences, and such important outliers – all of which bear their share of the European immigration challenge – evidence of other, more conjunctural forces of e.g. government ideology or coalitional politics? Or do they, after all, continue to reflect institutional and discursive path dependencies of national traditions and public philosophies of integration? Moreover, do further differences come into view when we investigate the turn to citizen-making in other fields, beyond the typical focus in the literature on integration requirements for entry, permanent residence and naturalization?

This issue seeks to elucidate such questions through a series of tightly matched comparative studies across a range of policy areas of a distinct European region, Scandinavia. As small and open countries Sweden, Norway, and Denmark face similar exposures to economic globalization. They share histories of international humanitarianism and staunch support of human rights conventions. Each cherishes national identities and public cultures of pride in social equality, promotion of women’s and children’s rights, and culturally liberated life styles. These accomplishments rest on the institutions of comprehensive welfare states of much the same type, and with much the same structural vulnerabilities – in Norway, particularly after recent setbacks of the oil economy. All three countries have based their large, still very redistributive public sectors on high labour market participation rates and tax payment capacity of both men and women. All are concerned about maintaining high levels of social equality and trust as elements of societal cohesion. These strong institutional, structural, and ideational similarities make them some of the most likely candidates to converge *if* the liberal convergence thesis is correct that global and European pressures external to the welfare state are the main drivers of national integration policy.

Yet, elites and large segments of electorates view the challenge of immigration and integration quite differently. If Sweden’s resistance towards even minimally demanding integration policies, at least at the gates of entry, residence, and citizenship – and as we shall see also other fields of integration – continues to perplex international commentators (Borevi, 2014), so does neighboring Denmark’s opposite trajectory. In this country, the late nineties departure from a common Scandinavian citizenship regime (Ersbøll, 2005) has been followed by ever tightened restrictions over the last two decades, with open public espousal by even mainstream politicians of nationalist cultural assimilation (Mouritsen & Olsen, 2013). Both countries’ politicians and publics view the other’s policies and discourse with increasing hostility. And Norway’s in-between course, previously inspired by Sweden, now leaning towards Denmark, provides an interesting example of normative cross-pressure – between concerns with cultural cohesion and pessimism concerning the will and ability of Muslim immigrants to adapt (as in Denmark), and a strong ideology of human rights and equal treatment (as in Sweden) (Brochmann & Hagelund, 2012).

Three of the articles in this special issue use Scandinavian countries to test the liberal convergence thesis in three policy areas outside the naturalization trajectory: labour market activation (Breidahl), family reunification (Bech, Borevi & Mouritsen) and the school (Fernández & Jensen). The fourth article (Simonsen) looks at the actual impact of civic conditioning on immigrants’ feelings of host national belonging, testing whether one of the rationales behind much restrictive naturalization policy withstands empirical scrutiny.

To assess the descriptive and explanatory arguments involved in the convergence thesis, some conceptual groundwork is called for. We must clarify what convergence means in the first place and what it *is* that supposedly converges. The next section discusses convergence in relation to the two concepts of civic integration and the civic turn. The following two sections apply these discussions to the Scandinavian cases and briefly introduce the two most central kinds of explanations for how integration policy has developed in Scandinavia: national philosophies and party-political dynamics. Before concluding, we discuss whether civic conditioning actually works.

## Civic integration, the civic turn and convergence

To assess and meaningfully talk about convergence, we need a clear understanding of what civic integration implies and, consequently, how we might measure the claim that countries are converging towards it. Is it a specific tool box of policy instruments to condition immigrants’ access to various legal statuses on meeting certain requirements or is it a perfectionist liberal, non-nationalistic philosophy of integration – or is it both?

Scholars doing increasingly sophisticated comparative work on citizenship acquisition, integration programs and tests tend to employ the term to denote a set of particular policies (Goodman, 2010; Howard, 2009; Koopmans, Michalowski, & Waibel, 2012). Others, notably Joppke himself and scholars debating whether or not multiculturalism and civic nationalism are either dead or “rebalanced” (Meer & Modood, 2009) and old school ethno-cultural nation-building discredited (Kostakopoulou, 2010), tend to see the term as an ideological reorientation of public philosophies as well as a common shift ‘from rights to duties’ in the dominant ideas about how immigrant integration is best promoted via state policies (e.g. Joppke, 2007, 2010). How and whether we actually assess claims of convergence depends on which conceptualization of civic integration we select. Moreover, as the following will show, defining it only in terms of policy instruments appears unsustainable while defining it in terms of a public philosophy implies that the field has had a too narrow policy focus.

Following the *first* understanding of civic integration as a particular set of policy instruments that conditions access to various legal statuses, it is clear that most European countries have introduced, since the nineties, formalized integration requirements pertaining to language, knowledge, and employment conditioning access to entry, permanent residence, citizenship, and, in some cases family reunification. Yet, the trouble with focusing exclusively on certain policy instruments is, first, that they can be designed in different ways and embody a range of different intentions, which cannot simply be ordered according to degree of restrictiveness (Goodman, 2012). Consequently, such analyses, even if they show policy convergence, cannot be used to argue that states are moving away from different national philosophies or nationalism towards more similar ideas about what integration is and entails. Second, with a restricted focus on civic integration requirements it arguably makes little sense even to talk of convergence. As Goodman (2010, 2014) demonstrates, before the introduction of such instruments in the mid-to-late 1990s, most countries had no formalized civic integration requirements attached to permanent residence and citizenship. Hence, the turn was not a move towards increasing similarity, but indeed *a turn*. European countries, in fact, were highly similar in *not* having such requirements. Therefore, at most, a move took place from one type of similarity to another – except that the significant variation in requirements more appropriately suggests divergence, and above all establishment of an independent policy area where none existed before.

Finally, and perhaps more crucial, we should critically discuss why certain demands and requirements are defined as civic integrationist and not others, and consequently how indexes to measure policies across countries are designed. Thus, with a narrower understanding of civic integration as a certain set of integration requirements, we run the risk of losing sight of *why* – i.e., according to which selection criteria – these requirements and indicators were chosen in the first place. In other words, in order to define the criteria for this particular *kind* of policy, we need to ask what it seeks to achieve. Yet, when we begin to define criteria in terms of the intent behind the policies we venture into the realm of ideas. Here, it becomes impossible to define a fixed list of policies, or policy areas for that matter, because, as already noted, a given policy can embody different intentions in different contexts. If civic integration policies reflect a new engagement across states to actively make immigrants into citizens, we should expect that other policies – besides the usually mentioned tests, contracts, courses and oaths – can be part of this development. Moreover, to the extent that variation exists across states, about what qualities and virtues constitute a “good citizen”, what counts as civic integration policies may even be country-specific.^2^ Along this line, Bech, Borevi and Mouritsen (2017) argue that the lack of measures of economic and age requirements in Goodman’s CIVIX index is a flaw in relation to family reunification, as such requirements, at least in a Scandinavian context, are closely connected to notions of civic maturity and self-sufficiency.

Conversely, if we understand the civic integrationist turn along the *second* line, as convergence mainly around a broader (neo)liberal, non-nationalistic philosophy of integration, within and between states, we should use other criteria for assessing the convergence thesis. Specifically, we must investigate whether intentions behind changing policies converge on a civic integrationist philosophy as distinct from national model-varieties of either cultural nationalist or multicultural rationales. This has two important implications. First, refuting the convergence thesis require more than simply demonstrating strong policy differences between countries – in the way e.g. Koopmans et al. (2012) arguably do. Ideational convergence on the level of public philosophy need not result in significant policy convergence since such ideas pass through national structures, institutions, agendas, and power dynamics before they translate into policy solutions. Second, an analysis of convergence cannot limit itself to a specific set of policies, because we cannot assume that states or governments draw similar policy conclusions from the same ideas. A public philosophy in and by itself is too abstract to provide clear-cut answers to which policy solutions serve its vision best (Mehta, 2010). Although a certain public philosophy cannot be paired with just anything, it only works as a meta-heuristic to narrow down the field of legitimate policy solutions. Consequently, different public philosophies may overlap in the kind of policies they enable, especially when we also remember that an integration requirement may express more than one intention. The upshot of all this is that we must always carefully trace the impact of philosophical ideas on policy instead of assuming that certain policies necessarily embody certain ideas (Daigneault, 2013).

A second, but related, point regarding public philosophies of integration – be it civic integration or some nationally specific kind – is that we should expect them to have an effect on all policies, which attempt to further immigrant integration – not just integration requirements for entry, permanent residence and naturalization. Consequently, analyses which argue that convergence has taken place – or dispute it – should expand their focus beyond the citizenship trajectory. Only few studies have taken this road so far. This special issue tries to do so by analyzing labour market policy, family reunification rules and school policy. Indeed, as argued by Fernández and Jensen (2017), the way that a public philosophy narrows down the range of legitimate policy solutions has just as much to do with how it configures policy areas as *relevant* for integration as with the policies employed within particular integration policy areas such as naturalization rules or citizenship education.

With this ideational perspective on convergence, we cannot assume, for example, that Sweden has not been impacted by the civic integrationist turn, just because it has no formalized integration requirements regarding language, knowledge or employment. An ideational change may have had different policy implications than in other countries.

Rather than ‘civic integration’, the title of this introduction and the special issue uses the term ‘civic turn’. We employ it to avoid the confusions that surround ‘civic integration’ – and to emphasize that, indeed, an *ideational turn* is visible while keeping an open mind about convergence or divergence regarding both ideas and policy. Yes, states increasingly emphasize civic culture or ‘good citizenship’ as modes of integrating culturally plural societies. However, citizenship, its universalistic core notwithstanding, is also a cultural phenomenon, which reflects different national trajectories and historical path-dependencies (as in historical sociology, e.g. Turner, 1990) and is susceptible to ideological contestations *within* countries. Hence, the notion of *liberal* convergence ignores that some instruments and policies, and certainly the national discourses legitimizing them, are hardly all liberal (or liberal in the same sense: some concern political liberal values, others neo-liberal economic self-reliance), but in some cases more “republican” or “instrumental nationalist” (Mouritsen, 2013).

In conclusion, Western European countries are indeed, in a broad sense, moving towards the universalistic corner of Koopmans, Statham, Giugni, and Passy’s (2005) well-known diagram. But the way they do so arguably reproduces old differences, restated within particular national vocabularies of civic nationalism, and leave many policies and institutions of immigrant incorporation in place.

## Comparing the Scandinavian countries

Sweden, Denmark and Norway are ideal candidates for a ‘most similar’ comparative case study. They are all small and open welfare state economies, built around similar comprehensive, universal welfare state ideology and organization (Esping-Andersen, 1990), subjecting them to similar financial pressures from the migration of low-skilled labour and the globalization of production (Andersen, 2004). Despite these similarities, the countries have approached immigrant integration quite differently, and these differences have increased in recent decades (Jensen, 2016; Djuve & Kavli, 2007). Hence, it is worthwhile to study these countries to further our understanding of the nature of the civic turn and its driving forces.

*First*, such comparisons help us show and assess the point made above, that different definitions and measures of civic integration may yield different conclusions in terms of how to characterize diverse national policy developments. In all indexes of (civic) integration policy, regardless of indicators, Denmark and Sweden are at opposite ends, Sweden in the permissive extreme and Denmark in the restrictive one. When included, Norway is typically placed closer to the middle. Denmark has strict economic, language and knowledge requirements with programs and tests for both permanent residence and naturalization. Norway has a mandatory introduction program and recently also a relatively soft language and knowledge tests for naturalization. Sweden still has no such integration requirements for permanent residence or naturalization. As they take into account the arguments behind policy changes, articles in this issue provide a more nuanced characterization of the civic turn in immigrant integration policy than those of the index studies, including the conclusion that Sweden, indeed, may also be part of it.^3^

*Second*, Scandinavian comparisons help us explore the explanatory power of public philosophies and national models in combination with factors highlighted in literatures on political power relations and party-political dynamics. Similarities in the countries’ welfare state system and economic situation allows us to exclude some of the external variables mentioned in the literature. If *external pressures* were main drivers of policy change, as in the liberal convergence thesis, convergence would be *particularly likely* between Scandinavian welfare states, which are similarly vulnerable to the structural squeeze of global competition and immigration because of their comparatively strong redistributive schemes and high wages.

Also, the countries remain relatively similar when it comes to public opinion on immigration and cultural diversity (Green-Pedersen & Krogstrup, 2008). Studies using data from the European Social Survey show that, although the Swedish public generally displays a somewhat more positive attitude towards immigration and support for equal rights, the Danish and Norwegian publics typically cluster with Sweden when compared to other European countries (Gorodzeisky & Semyonov, 2009; Nagayoshi & Hjerm, 2015; Sides & Citrin, 2007).

However, the three countries diverge on two internal factors, each offering (competing) explanatory accounts: national identities and public philosophies; and party-political dynamics.

We argue, as do each of the articles in this special issue, that the Scandinavian countries, despite their similarities, represent significantly different national identity constructions. While the universalist welfare state is crucial to each country’s national self-image (Berggren & Trägårdh, 2006; Mouritsen, 2006; Mouritsen & Olsen, 2013; Stråth, 2000), and while all countries depend on high levels of trust and solidarity, social mobility, high labour market participation and high taxation levels (Andersen, 2004), each country has its own understanding of *how* social cohesion and welfare state sustainability comes about.

In Denmark, a society-centered and bottom-up perspective is the predominant, which sees (pre)existing cultural homogeneity as indispensable for the welfare state and maintains a ‘populist’ view of political sovereignty, where politicians should respond to, and respect the people, and where legislation to a lesser extent reflects preparatory commissions and expert advice (Jensen, 2014). In Sweden, a more state-centred and top-down approach prevails, whereby the modernizing welfare state promotes social inclusion and integrates society through equal treatment (Borevi, 2017). Politicians are more likely to see their role as one of leading the people and propose legislation in accordance with these guiding norms, and with social science emanating from expert commissions. Norway occupies an ambivalent, perhaps even confused, middle ground, which mixes strong concern with cultural and identitarian cohesion – but often filtered through expert commissions as ‘instrumental’ sociological arguments (rather than Denmark’s more populist political culture) – with equal treatment and human rights concerns, which are closer to Sweden.

Different historical experiences no doubt contribute to these ideational variations. Despite rather similar histories of ethnic homogeneity, immigration, and dominant Lutheran state churches, the countries had different experiences with mass emigration to the US in the early 20^th^ century (nearly 1.5 million Swedes emigrated, only some 400.000 from Denmark) as well as with the size and timing of post-war immigration, which was larger and started earlier in Sweden compared to Denmark and Norway. This arguably contributed to the emergence and reinforcement of different path dependencies in immigrant integration policy (Borevi, 2012, 2017; Bengtsson & Borevi, 2015). So did, possibly, the fact that only Norway and Sweden had ‘internal’ territorial aboriginal minorities (the Sami and Finish populations). Denmark, by comparison, had colonies (Greenland and the Faroe Islands) and a small German-speaking minority in Schleswig. Finally, Sweden’s neutrality during the Second World War (where Norway and Denmark became occupied) may have induced more sustained questioning of its own past (e.g., eugenics in the thirties) and impetus towards anti-racism than in the other two countries.

The different reasoning of Danish, Swedish, and Norwegian policy-makers in matters of immigrant integration may reflect such variations of national self-understanding. The most obvious example, which is not discussed in this special issue (but see Jensen, 2016; Brochmann & Seland, 2010), may be seen in rules and arguments surrounding citizenship acquisition in the three countries. Denmark’s *Sonderweg* departure since 2001 from a common Scandinavian naturalization regime towards increasingly heavy-handed conditionalities, and easier loss of nationality (Ersbøll, 2016; Mouritsen, 2013), contrasts starkly with Sweden’s continuously very liberal, lightly conditioned regime. Norway’s nationality law characteristically falls somewhere in the middle, with both restrictive and liberal elements, including much more lenient language and knowledge requirements.

Where Danish debates on naturalization remain highly politicized, tied to issues of deservingness, cultural assimilation, security, and ideas about citizenship as something sacred, which *should* be difficult (Mouritsen & Olsen, 2013), Norwegian debates have been less protracted and discourses less harsh. They have centered on concern that new citizens should be able to belong and have undivided political loyalty – hence Norway’s continuing resistance to dual citizenship – as well the vulnerability of Norway as a small language community. Yet, despite introduction of a light language requirement, an oath of allegiance, and mandatory participation in an integration program (without tests), citizenship is now, unlike previously and in sharp contrast to Denmark, a legal entitlement (Midtbøen, 2015). Sweden, characteristically, has had no debate to speak of, and citizenship remains demonstrably void of national symbolism, with naturalization being an easy, largely administrative affair (Bernitz & Bernitz, 2005).

Turning to party political factors, we also find important differences. First, while Denmark and Norway have had a long experience of electorally quite successful populist right wing parties, this is a more recent phenomenon in Sweden. In both Denmark and Norway, a populist right-wing party – named the Progress Party in both countries – came into parliament in 1973. The Norwegian Progress Party (*Fremskrittspartiet*), increasingly successful, since 1997 received between 14 and 23 per cent of the vote and is now part of the Norwegian government. Until today, their influence on integration policies has been curbed by a strong, partly strategic, political norm of broad consensus on such issues (Bale, Green-Pedersen, Krouwel, Luther, & Sitter, 2009). The Danish Progress Party (*Fremskridtspartiet*) had the opposite experience with early success but lower polls in the 1990s. In 1995 the Danish People’s Party (*Dansk Folkeparti*), which broke away from the Progress Party in 1995, emerged as the new major populist right-wing party. It has exercised significant influence on Danish immigration and citizenship policies, particularly because the 2001-11 Conservative-Liberal government depended on them for its parliamentary majority in most cases. In Sweden, during a short period 1991–1994, a populist right-wing party – New Democracy (*Ny Demokrati*) – was for the first time represented in parliament but only since 2010 have a new populist right-wing party – the Sweden Democrats (*Sverigedemokraterna*) – gained a more continuous representation. Contrary to Denmark and Norway, the strategy of the mainstream parties has been to isolate the Sweden Democrats, which means that, at least in the short run, its parliamentary success has not translated in stricter policies, but rather the contrary, as all other parties actively dissociated themselves from this party.

Different conditions for centre-right coalitions have also been used to explain policy variations (Green-Pedersen & Krogstrup, 2008; Green-Pedersen & Odmalm, 2008). In Denmark, centre-right parties only politicized integration issues and pursued restrictive policies when the centrist Social Liberal Party was no longer seen as a viable government partner (in 1993). In Sweden, by contrast, the Conservative party (*Moderaterna*) has had an incentive to take a moderate approach, as its centre-right coalition partners (the Liberals, the Agrarian Centre Party and (since 1991) the Christian Democrats) all had outspoken pro-immigration profiles. In Norway, centre-right parties chose a strategy of defusing integration issues in concert with the centre-left (Bale et al., 2009), creating a strong norm for consensus in issues of integration.

We contend that explanations based on party political dynamics and power relations do not exclude explanations based on national path dependencies of political culture and national self-understanding. Indeed, in both Sweden and Denmark, conceptions of democracy, nationhood, and the relation between state and people may reinforce party dynamics. As already touched upon, it is unfruitful to conceptualize philosophies of integration as tight-knit paradigms, encompassing particular policy solutions, which thereby circumscribe politics by definition. Instead, a dominant public philosophy of integration sets the boundaries of public disagreement while leaving room for party-political dynamics to simultaneously impact policy decisions. As such, its effect regards *how far* party politics may shift policies in one or the other direction. Hence, how centre-left and centre-right parties respond to the success of populist right-wing parties may also be influenced by what kind of challenge such a party constitute to the dominant public philosophy of integration. In Denmark’s populist-communitarian context, it has been less of a challenge than it remains in modernist-liberal Sweden.

## The three comparative case studies in this special issue

Three of the studies in this special issue go beyond the naturalization trajectory and focus on labour market activation policies in the three countries, the rules for family reunification in all three countries, and citizenship education in Denmark and Sweden, respectively. The main question is whether party-political dynamics and philosophical ideas are indeed similar across these different areas of integration policy, and whether these areas display the same patterns as those seen within the politics of permanent residence and naturalization. To what extent, if at all, is there a dominating philosophical and political pattern in the integration politics of these three countries?

In their article, *Férnandez and Jensen* show that within citizenship education policy Denmark and Sweden has undergone a similar divergence starting in the 1990s, as was the case within naturalization policy. Behind this divergence, they argue, are two very different, rather stable and consensual public philosophies. A Danish monocultural philosophy, in which “assimilation is viewed normatively and causally as a precondition of equal inclusion and opportunity” and a Swedish philosophy that seeks an “equilibrium between pluralism and universalism” and renders “equal inclusion and opportunity contingent on the ability of each, especially the majority, to recognize the others.” Interestingly, they also find that Swedish school politics have been highly concerned with how to form good, liberal-democratic citizens in response to international migration, concluding that Sweden has indeed taken part in the civic turn.

*Bech, Borevi and Mouritsen* argue that family reunification policies constitute one important, yet underexplored, field of civic integration. The article contributes to discussions about the definition and measurement of civic integration in response to the CIVIX index created by Goodman (2010, 2014). It makes two specific arguments. First, that economic demands and age-requirements for family reunification is part of a larger civic integration trend pertaining to ideas and norms of civic maturity, gender equality and individual autonomy. Second, that we must take into account how family migration involves a ‘double conditionality’ whereby integration requirements target *both* the civic deservingness of the resident sponsor *and* the civic integration potential of an incoming family member. Following this broader view of civic integration or indeed *civic selection* – and taking into account the argumentative structures surrounding policy change (and non-change) – the article systematically describes and compares the trajectories of family migration policies in the three Scandinavian countries since the late 1990 including the most recent changes. Policy responses remain remarkably diverse, and in Sweden and Denmark’s cases non-converging, reflecting national philosophies of integration. However, the article also documents a growing salience of a controlling-the-number logic in all three countries, particularly in the wake of 2015 refugee crisis, which increasingly crowds out the politics of civic integration.

*Breidahl* contributes with a comparative study of labour market activation policies in Denmark, Sweden and Norway. In this integration policy area, rather than divergence, important similarities come into view. All Scandinavian countries have introductory programs for new-arrivals with strong emphasis on conditionality, although Sweden does not tie participation in the programs to permanent residence or citizenship. Breidahl argues that similar, strong and rooted norms of state involvement and employment capacity, closely connected to the functionality of the universal welfare state, exist in all three countries. However, she concludes that the “strong interconnection between activation policies and the civic turn seems to be exceptional to the Scandinavian welfare states and should not be interpreted as a retreat from national models”. Instead, it is a shared feature of their respective national models, originating in the fiscal requirement of the universal welfare state for high unemployment.

Overall, these three studies, in conjunction with existing ones on entry and naturalization, demonstrate how the different public philosophies in the three countries configure *which* policy areas become vehicles of civic integration as well as *how* civic integration is approached within each of them.

## Does civic integration policies work?

Evaluating whether civic integration policies actually work is difficult. Not only is high quality data scarce but there are different ideas about the very goal of civic integration policies making it uncertain which outcomes to investigate. In addition, we need to know which policies actually count as civic integration, before evaluating their effectiveness.

The measures mostly associated with civic integration – integration requirements for entry, permanent residence and naturalization – are often justified as ways to incentivize and motivate immigrants to acquire certain capacities, attitudes and knowledge that allows them to participate in the labor market, civil society and democracy. However, as many studies show, integration requirements also serve as a civic screening mechanism to keep out those (potential) immigrants who are more difficult to integrate (Goodman, 2011; Groenendijk, 2011; Van Oers, Ersbøll, & Kostakopoulou, 2010). Finally, civic integration policies may have *symbolic* goals, e.g. to signal to host populations, either that the competence and ability of governments to control the country’s borders, or their concern to maintain and strengthen cultural cohesion and national identity (Goodman & Wright, 2015, p. 1891; Permoser, 2012).

Accordingly, the question of whether these typical civic integration policies work can be formulated (at least) in three ways: (1) does civic conditioning further the integration of immigrants?; (2) do civic integration policies screen for less easily integratable immigrants?; (3) do policies reinforce citizens’ own narratives of nationhood? These goals reflect the political reasoning of Western states generally, although, from a normative point of view, it is debatable that states may legitimately use any integration policy, of any degree of restrictiveness, to further them (e.g., Carens, 2013; Miller, 2008).

To our knowledge, the two last goals of civic integration policies have received little empirical attention. The immediate gatekeeping effects, in terms of pass rates for tests, naturalization rates, and the type and numbers of immigrants arriving has been better researched (e.g., Dronkers & Vink, 2012; Van Oers, 2013). A Danish evaluation report showed that 40 percent of the applicants in 2008 were denied citizenship on grounds of language insufficiency (Ersbøll & Gravesen, 2010). However, no one has investigated whether integration requirements actually select the immigrants most likely to (further) integrate.

Some efforts have been devoted to the first question above. Goodman and Wright (2015) assess the impact of civic integration policies on immigrants’ social integration and report null-findings – policies seem to have little impact, either positively or negatively, on immigrants’ self-reported financial well-being and levels of social trust. Ersanilli and Koopmans (2011), in a comparison of Turkish guest workers in France, Germany and the Netherlands, find linguistic and social assimilation to occur more when integration requirements condition access to rights – although the effect is small.

In light of such research lacunae, Simonsen, in this special issue, importantly advances our knowledge in her study on the impact of citizenship policies on immigrants’ sense of host national belonging. Taking her theoretical point of departure in the assumption that naturalization not only provides access to certain rights (“the legal economy”), but also importantly signals full membership of the host nation (“the symbolic economy”), Simonsen sets out to assess the importance of “policy signals” for immigrants’ self-reported feelings of host national belonging. The article can be read as a theoretical development and empirical test of the underlying logics (or intended mechanisms) expressed in the civic integration discourse, i.e. “that civic conditioning is conducive to integration”. However, Simonsen adds a symbolic mechanism to this thesis. Given that citizenship is very exclusive due to demanding civic requirements, we would expect someone who attains this status to develop a stronger host nation attachment or identification compared to someone who naturalizes into a country with a more liberal “open door” citizenship regime. The study results in a null finding. By themselves, policy signals (either strict or liberal) have no effect on immigrants’ sense of belonging. The study does “support the idea that citizenship matters for feelings of belonging, but only when it also matters for host nationals in their perceptions of who belongs”. It seems to be the general attitude of the public, not policies, which matters for cultivation of national identification.

## Conclusion

It is difficult to counter the claim that integration politics in the West has given way to a liberal-democratic semantic and focus on good citizenship while overtly ethno-nationalist reasoning has been delegitimized. However, we argue that the study of how civic integration policies develop must be attentive to how different understandings of ‘civicness’ dominates in different countries in ways that tie in with conceptions of nationhood, the ideational boundaries of party politics, and the reactions of mainstream political parties to successful populist right-wing parties. Moreover, the field should look beyond the citizenship trajectory to other areas of integration policy to gain a deeper awareness of civic integration in its different forms.

The Scandinavian countries are good examples. On the surface political parties seem to subscribe to similar notions of good citizenship and social cohesion, but underneath they diverge strongly as to what maintaining strong welfare states implies in terms of the efforts and obligations of immigrants and citizens, and as to where the state should place its effort. They have quite different ideas about the socialization process through which immigrants must pass in order to integrate, and what motivates immigrants to do so. In other words, studies must be aware of the empirical assumptions political actors make use of; their notions of what works and why (Jensen, 2014). Especially, since they have little empirical knowledge, based on social science research, to guide their claims – the few studies that do exist suggest either no or minimal policy effects – these should be viewed as ideological. Scholars in the field must intensify their efforts to study the actual effects of civic integration policies, and, by providing such factual grounding, they must help shelter policy developments in the field from the worst entrapments of ‘common sense’ ideological illusions about what works and why.

## Footnotes

1 Within Joppke’s own convergence reading lurks, paradoxically, variations between Rawlsian (UK), perfectionist republican-liberal and form-of-life liberal (France, Netherlands), and “repressive” *Gesinnung*-liberalisms (some conservative *Länder* in Germany) (Joppke, 2009).

2 This of course potentially constitute a fundamental problem for index construction as such, since it affects the comparability of country scores.

3 To the extent the civic turn denotes a ‘retreat from multiculturalism’ – a shift in focus from concerns of the collective identities of immigrants to a preoccupation of defining and strengthening the host national identity – then policy developments in Sweden, at least since the early 1990s, may well be portrayed as part of this trend (Borevi, 2010, 2014).

## References

[CR1] Andersen TM (2004). Challenges to the Scandinavian welfare model. European Journal of Political Economy.

[CR2] Bale T, Green-Pedersen C, Krouwel A, Luther KR, Sitter N (2009). If You Can’t Beat Them, Join Them? Explaining Social Democratic Responses to the Challenge from the Populist Radical Right in Western Europe. Political Studies.

[CR3] Bech, E. C., Borevi, K. & Mouritsen, P. (2017). A ‘civic turn’ in Scandinavian family migration policies? Comparing Denmark, Norway and Sweden. *Comparative Migration Studies, 5*, 7. 10.1186/s40878-016-0046-7 PMC5331100 28303235

[CR4] Bengtsson, B., & Borevi, K. (2015). Mångfaldens vägskäl. Om integrationspolitikens stigberoende [Critical junctures of diversity. On path dependence in immigrant integration policies]. In B. Bengtsson, G. Myrberg, & R. Andersson (Eds.), *Mångfaldens dilemman. Integrationspolitik och medborgarskap* (pp. 17–40). Malmö: Gleerups förlag.

[CR5] Berggren, H., & Trägårdh, L. (2006). *Är svensken människa? Gemenskap och oberoende i det moderna Sverige [Is the Swede Human? Radical Individualism in the Land of Social Solidarity].* Stockholm: Norstedt.

[CR6] Bernitz HL, Bernitz H, Bauböck R, Ersbøll E, Groenendijk K, Waldrauch H (2005). Sweden. Acquisition and Loss of Nationality. Policies and Trends in 15 European Countries. Volume 2: Country Analyses.

[CR7] Bonjour S, Lettinga D (2012). Political Debates on Islamic Headscarves and Civic Integration Abroad in France and the Netherlands: What Can Models Explain?. Journal of Immigrant & Refugee Studies.

[CR8] Borevi K, Bengtsson B, Strömblad P, Bay A (2010). Dimensions of citizenship: European integration policies from a Scandinavian perspective. Diversity, inclusion and citizenship in Scandinavia.

[CR9] Borevi K, Brochmann G, Hagelund A (2012). Sweden: the Flagship of Multiculturalism. Immigration Policy and the Scandinavian Welfare State 1945–2010.

[CR10] Borevi K (2014). Multiculturalism and welfare state integration: Swedish model path dependency. Identities: Global Studies in Culture and Power.

[CR11] Borevi K, Banting K, Kymlicka W (2017). Diversity and Solidarity in Denmark and Sweden. Strains of Commitment: The Political Sources of Solidarity in Diverse Societies.

[CR12] Brochmann G, Hagelund A, Brochmann G, Hagelund A (2012). Norway: The Land of Golden Mean. Immigration Policy and the Scandinavian Welfare State 1945–2010.

[CR13] Brochmann G, Seland I (2010). Citizenship policies and ideas of nationhood in Scandinavia. Citizenship Studies.

[CR14] Carens J (2013). The Ethics of Immigration.

[CR15] Daigneault P-M (2013). Reassessing the concept of policy paradigm: aligning ontology and methodology in policy studies. Journal of European Public Policy.

[CR16] Djuve, A. B., & Kavli, H. C. (2007). *Integrering i Danmark, Sverige og Norge: Felles utfordringer – like løsninger? [Integration in Denmark, Sweden and Norway: Common challenges – similar solutions?]. *København: Nordisk Ministerråd.

[CR17] Dronkers J, Vink MP (2012). Explaining access to citizenship in Europe: How citizenship policies affect naturalization rates. European Union Politics.

[CR18] Ersanilli E, Koopmans R (2011). Do Immigrant Integration Policies Matter? A Three-Country Comparison among Turkish Immigrants. West European Politics.

[CR19] Ersbøll E, Bauböck R, Ersbøll E, Groenendijk K, Waldrauch H (2005). Denmark. Acquisition and Loss of Nationality. Policies and Trends in 15 European Countries. Volume 2: Country Analyses.

[CR20] Ersbøll E, Nunez SC, de Groot G-R (2016). Loss of nationality in the Nordic countries. European Citizenship at the Crossroads: The Role of the European Union on Loss and Acquisition of Nationality.

[CR21] Ersbøll E, Gravesen LK (2010). Country Report Denmark. Report from The INTEC Project: Integration and Naturalisation Tests: The New Way to European Citizenship.

[CR22] Esping-Andersen G (1990). The Three Worlds of Welfare Capitalism.

[CR23] Fernández, C. & Jensen K. K. (2017). The civic integrationist turn in Danish and Swedish school politics. *Comparative Migration Studies, 5*, 5. 10.1186/s40878-017-0049-z PMC5331097 28303234

[CR24] Goodman SW (2010). Integration requirements for integration’s sake? Identifying, categorizing and comparing civic integration policies. Journal of Ethnic and Migration Studies.

[CR25] Goodman SW (2011). Controlling Immigration through Language and Country Knowledge Requirements. West European Politics.

[CR26] Goodman SW (2012). Fortifying Citizenship: Policy Strategies for Civic Integration in Western Europe. World Politics.

[CR27] Goodman SW (2014). Immigration and Membership Politics in Western Europe.

[CR28] Goodman SW, Wright M (2015). Does Mandatory Integration Matter? Effects of Civic Requirements on Immigrant Socio-economic and Political Outcomes. Journal of Ethnic and Migration Studies.

[CR29] Gorodzeisky A, Semyonov M (2009). Terms of exclusion: public views towards admission and allocation of rights to immigrants in European countries. Ethnic and Racial Studies.

[CR30] Green SO (2007). Divergent traditions, converging responses: immigration and integration policy in the UK and Germany. German Politics.

[CR31] Green-Pedersen C, Krogstrup J (2008). Immigration as a political issue in Denmark and Sweden. European Journal of Political Research.

[CR32] Green-Pedersen C, Odmalm P (2008). Going different ways? Right-wing parties and the immigrant issue in Denmark and Sweden. Journal of European Public Policy.

[CR33] Groenendijk K (2011). Pre-departure Integration Strategies in the European Union: Integration or Immigration Policy?. European Journal of Migration and Law.

[CR34] Howard MM (2009). The Politics of Citizenship in Europe.

[CR35] Jensen, K. K. (2016). *Scandinavian Immigrant Integration Politics: Varieties of the Civic Turn*. PhD dissertation, Aarhus University. Aarhus.

[CR36] Jensen KK (2014). What can and cannot be willed: how politicians talk about national identity and immigrants. Nations and Nationalism.

[CR37] Joppke C (2007). Beyond national models: Civic integration policies for immigrants in Western Europe. West European Politics.

[CR38] Joppke C (2009). Veil – Mirror of Identity.

[CR39] Joppke C (2010). Citizenship and Immigration.

[CR40] Koopmans, R., Michalowski, I., & Waibel, S. (2012). Citizenship rights for immigrants: National political processes and cross-national convergence in Western Europe, 1980–2008. *American Journal of Sociology, 117*(4), 1202–1245. 10.1086/662707 22594120

[CR41] Koopmans R, Statham P, Giugni M, Passy F (2005). Contested Citizenship: Immigration and Cultural Diversity.

[CR42] Kostakopoulou D (2010). The Anatomy of Civic Integration. Modern Law Review.

[CR43] Meer N, Modood T (2009). The Multicultural State We’re In: Muslims, “Multiculture” and the “Civic Re-balancing” of British Multiculturalism. Political Studies.

[CR44] Mehta J, Béland D, Cox RH (2010). The Varied Roles of Ideas in Politics. Ideas and Politics in Social Science Research.

[CR45] Midtbøen AH (2015). Citizenship, integration and the quest for social cohesion : nationality reform in the Scandinavian countries. Comparative Migration Studies.

[CR46] Miller D (2008). Immigrants, Nations, and Citizenship. Journal of Political Philosophy.

[CR47] Mouritsen, P. (2006). The Particular Universalism of a Nordic Civic Nation: Common Values, State Religion, and Islam in Danish Political Culture. In T. Modood, A. Triandafyllidou, & R. Zapata-Barrero (Eds.), *Multiculturalism, Muslims and Citizenship: A European Approach* (pp. 70–93). New York: Routledge.

[CR48] Mouritsen, P. (2013). The resilience of citizenship traditions: Civic integration in Germany, Great Britain and Denmark. *Ethnicities, 13*(1), 86–109.

[CR49] Mouritsen P, Olsen TV (2013). Denmark between Liberalism and Nationalism. Ethnic and Racial Studies.

[CR50] Müller J-W (2007). Is Europe Converging on Constitutional Patriotism? (And If So: Is It Justified?). Critical Review of International Social and Political Philosophy.

[CR51] Nagayoshi K, Hjerm M (2015). Anti-immigration attitudes in different welfare states: Do types of labor market policies matter?. International Journal of Comparative Sociology.

[CR52] Orgad L (2015). The Cultural Defense of Nations: A Liberal Theory of Majority Rights.

[CR53] Permoser JM (2012). Civic Integration as Symbolic Politics: Insights from Austria. European Journal of Migration and Law.

[CR54] Sides J, Citrin J (2007). European Opinion About Immigration: The Role of Identities, Interests and Information. British Journal of Political Science.

[CR55] Stråth B, Stråth B (2000). Poverty, Neutrality and Welfare: Three Key Concepts in the Modern Foundation of Sweden. Myth and Memory in the Construction of Community.

[CR56] Tebble A (2006). Exclusion for Democracy. Political Theory.

[CR57] Triadafilopoulos, T. (2011). Illiberal Means to Liberal Ends? Understanding Recent Immigrant Integration Policies in Europe. *Journal of Ethnic and Migration Studies, 37*(6), 861–880.

[CR58] Turner BS (1990). Outline of a Theory of Citizenship. Sociology.

[CR59] Van Oers, R. (2013). *Deserving Citizenship: Citizenship Tests in Germany, the Netherlands and the United Kingdom*. Leiden: Martinus Nijhoff Publishers.

[CR60] Van Oers, R., Ersbøll, E., & Kostakopoulou, D. (2010). Mapping the redefinition of belonging in Europe. In R. Van Oers, E. Ersbøll, & D. Kostakopoulou (Eds.), *Re-definition of Belonging? Language and Integration Tests in Europe* (pp. 307–331). Leiden, Boston: Martinus Nijhoff Publishers.

